# Treatment of Intracranial Hemorrhagic Lesions Associated With Jacobsen’s Syndrome

**DOI:** 10.7759/cureus.43486

**Published:** 2023-08-14

**Authors:** Michihiro Kurimoto

**Affiliations:** 1 Pediatric Neurosurgery, Aichi Children's Health and Medical Center, Obu, JPN

**Keywords:** blood testing, anti-coagulation, coagulation disorders, intracranial hemorrage, jacobsen's syndrome

## Abstract

Jacobsen's syndrome is a rare genetic disorder caused by deletion of the long arm of chromosome 11 (11q) and is characterized primarily by craniofacial dysmorphism, congenital heart defects, intellectual disability, Paris-Treussaud hemorrhagic disorder, structural renal defects, and immunodeficiency. Although the frequency of intracranial hemorrhage associated with Jacobsen's syndrome is low, it is recognized as an important prognostic factor. In this report, we describe a case of acute and chronic subdural hematoma that developed during anticoagulation therapy after cardiac surgery for congenital heart defects associated with Jacobsen's syndrome, making it difficult to decide on a treatment plan.

## Introduction

Hemorrhagic complications, mainly Paris-Treussaud hemorrhagic disorder, are well known in Jacobsen's syndrome [1,2]. Intracranial hemorrhage has been reported in a few cases in the past [3], and many of them are fatal [4]. In general, when a neurosurgeon is faced with a patient with an intracranial hemorrhagic lesion requiring anticoagulant therapy due to prior treatment, the surgeon must attempt to achieve maximum hemostasis and, if necessary, may decide to discontinue anticoagulant therapy, recognizing the risk of complications. In addition, although strict management must be maintained when anticoagulation therapy is used, which can be monitored by blood tests, there is always a risk of further complications due to coagulation abnormalities that cannot be proven by routine blood tests or vascular abnormalities that are not yet known.

In this report, we describe a case of acute subdural hematoma after surgery for cardiac disease associated with Jacobsen's syndrome, which was aggravated when anticoagulation therapy was resumed even after hematoma removal.

## Case presentation

The patient was a young male aged two years and 11 months (35 months), who had been delivered via emergency cesarean section because of fetal heart rate failure. A general physical examination revealed peculiar facial features, auricular hypoplasia, and overlapping fingers. Auscultation revealed gallop rhythm, and echocardiography revealed common artery stem disease, aortic arch transection, ventricular septal defect, aortic regurgitation (severe), mitral stenosis, hypoplastic left heart, and left superior vena cava remnant. A horseshoe kidney was also observed at a later examination, and G-banding test revealed a deletion of chromosome 11 long arm, and confirmed the diagnosis of Jacobsen's syndrome.

The following is a summary of the history of cardiac treatment. On day six, bilateral pulmonary artery strangulation was performed. At three months, aortic valvuloplasty, expanded aortic arch anastomosis, and palliative Rastelli surgery were performed. Preoperatively, the patient was pancytopenic and required granulocyte colony-stimulating factor (G-CSF) administration. Postoperatively, there was a pseudoaneurysm in the left ventricular outflow tract. At 12 months, aortic valve replacement was performed. At 20 and 26 months, a stent was placed in the right pulmonary artery for endovascular treatment. At 35 months, while hospitalized for endovascular treatment, the patient's level of consciousness decreased, and Neurosurgery consultation was sought.

**Figure 1:**
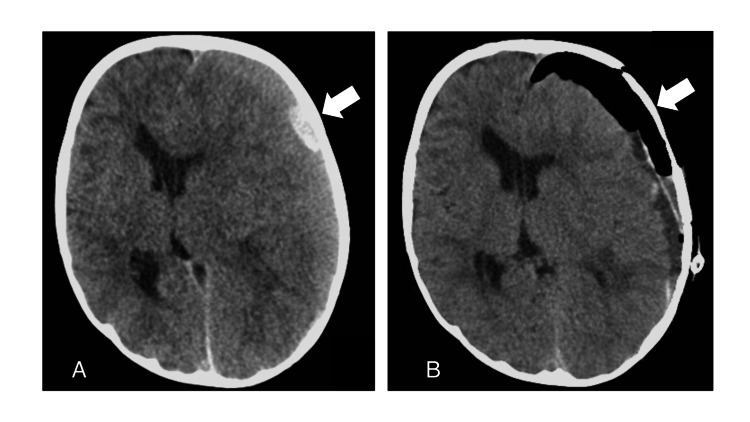
Preoperative and postoperative CT of the head (A) Preoperative head CT  showing acute and chronic subdural hematoma on the left side with midline shift; (B) Head CT just after hematoma removal surgery

Initial physical examination on day zero (on Neurosury consultation at 35 months) revealed a Glascow coma scale (GCS) score of E4V4M6, no pupil irregularity, and mild right hemiplegia. Head CT scan showed acute and chronic subdural hematoma on the left side with midline shift (Figure 1A). The patient had been on anticoagulation and antiplatelet therapy since aortic valve replacement (open heart surgery) at 11 months, and blood tests showed a low platelet count and severely prolonged INR of 5.6. Therefore, platelet transfusion and antagonistic treatment were performed first, followed by craniotomy to remove the hematoma on day one (Figure 1B), and the source of bleeding was presumed to be the Sylvian vein based on intraoperative findings. Pupillary discrepancy appeared preoperatively but improved soon postoperatively.

**Figure 2:**
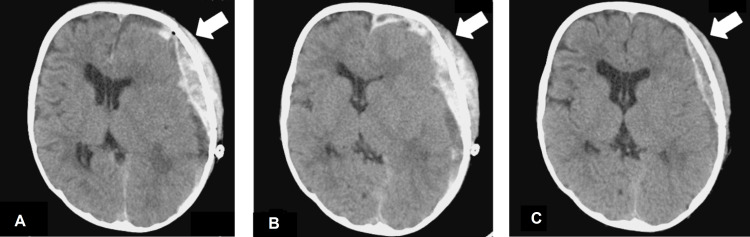
Head CT after resumption and re-discontinuation of anticoagulation (A) Head CT on day 7. Midline shift improved with a decrease in subdural hematoma; (B) Head CT on day 9. After anticoagulation therapy was resumed on postoperative day 7, the subdural hematoma gradually increased; (C) Head CT on day 22. Anticoagulation therapy was resumed on day 18, but no hemorrhagic complications were observed.

**Figure 3:**
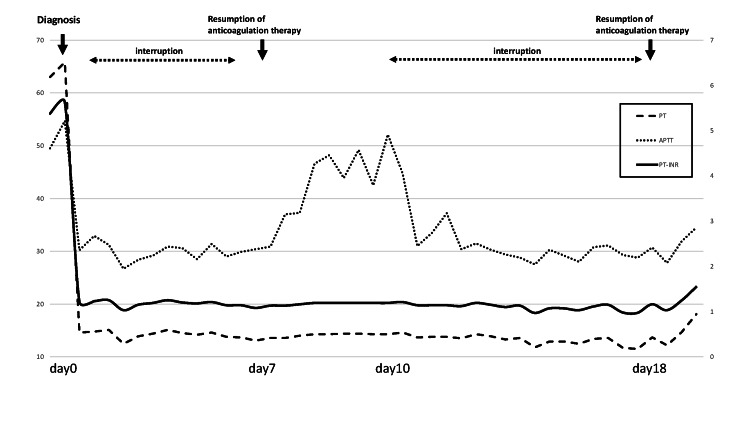
Trends in blood coagulation function tests from the time of admission to treatment and the duration of anticoagulation therapy

Postoperatively, the patient was followed up without anticoagulation or antiplatelet therapy, and the midline shift improved with a decrease in subdural hematoma by day seven (Figure 2A). During this period, platelet levels remained continuously low, and platelet transfusions were administered as needed. But after anticoagulation therapy was resumed on day seven, head CT on day nine showed an obviously increased subdural hematoma (Figure 2B). Therefore, after consultation between the cardiologist, cardiovascular surgeon, and intensivist, anticoagulation treatment had to be discontinued until complete hemostasis was achieved, although the risk of thrombotic complications increased. After discontinuation of anticoagulation treatment, the patient was followed up with careful observation of his general condition and neurological findings for 10 days, after which the subdural hematoma gradually decreased and midline shift improved. Anticoagulation therapy was resumed on day 18, but no hemorrhagic complications were observed on the head CT done on day 22 (Figure 2C). Figure 3 shows the timeline of trends in blood coagulation function tests.

## Discussion

Patients with Jacobsen's syndrome require great attention to systemic hemorrhagic complications, as exemplified by Paris-Trousseau hemorrhagic disorder. The pathophysiology may include abnormal hemostasis associated with thrombocytopenia and abnormalities in the coagulation function itself, and the possibility of falling into a paradox with the treatment of comorbidities when bleeding occurs should be fully considered [5]. In the present case, overzealous anticoagulation therapy was a possible factor in the onset of the disease, but the patient had obvious abnormalities in hemostatic function, including easy rebleeding despite a one-week withdrawal and hemostasis period.

In addition to common blood cell tests (platelet counts, prothrombin time, activated partial thromboplastin clotting time (aPTT)), other numerical visualizations of coagulation abnormalities include thromboelastography (TEG) and thromboelastometry (ROTEM) [6], measurement of von Willebrand factor (VWF) and Factor 13, and platelet aggregation capacity [7]. In this case, blood viscoelasticity was almost normal, vWFs were normal, factor 13 antigen and activity were mildly decreased, and platelet aggregation capacity was not performed because it is a research-based measurement. Based on the above results, there were no problems with comprehensive hemostatic function or secondary hemostatic function, and because of the possible influence of intracranial hemorrhage on factor 13 levels, it was not possible to quantify any abnormalities related to hemostatic function.

In such cases, we are often confused between the risk of thrombotic complications associated with prosthetic valve insertion and the need to treat bleeding complications. Guidelines for anticoagulation in postoperative patients following prosthetic valve insertion indicate that while the absence of anticoagulation is a risk for thrombotic complications, "inappropriate" anticoagulation can also be a risk for both thrombotic and hemorrhagic complications. Anticoagulant medications are also mentioned with warfarin as the first choice, but there is no clear recommendation in cases of coexisting congenital coagulation dysfunction or in the development of bleeding complications [8].

There have also been many reports on vascular lesions in Jacobsen's syndrome, and in the present case, complications such as dissection of the pulmonary artery and pseudoaneurysm formation in the left ventricular outflow tract, which do not commonly occur during cardiac surgery, were observed. In a previous report on intracranial hemorrhage, an intracranial aneurysm was found in one of six cases, and a review in the field of ophthalmology also reported the presence of vascular tortuosity in many cases [4,9].

We can take measures such as aggressive platelet transfusion for persistent thrombocytopenia in the perioperative period and delay the resumption of anticoagulation therapy as long as possible, but these measures are not expected to be effective. We hope that the pathogenesis of the disease will be elucidated, and that treatment guidelines will be formulated to deal with bleeding complications when they develop.

## Conclusions

The treatment of a case of intracranial hematoma associated with Jacobsen's syndrome is described in this report. It should be noted that there may be latent abnormalities that cannot be ascertained by general coagulation function measurements and may require temporary interruption of challenging anticoagulation therapy after surgical treatment. In addition, although the present case involved extraparenchymal hemorrhage, there have been reports of complications of intracranial vascular lesions, and attention to vascular vulnerability will be essential during the treatment of such lesions.
